# In Situ Regulation of Macrophage Polarization to Enhance Osseointegration Under Diabetic Conditions Using Injectable Silk/Sitagliptin Gel Scaffolds

**DOI:** 10.1002/advs.202002328

**Published:** 2020-12-16

**Authors:** Geng Xiang, Keyin Liu, Tianji Wang, Xiaofan Hu, Jing Wang, Zhiheng Gao, Wei Lei, Yafei Feng, Tiger H. Tao

**Affiliations:** ^1^ Department of Orthopedics Xijing Hospital The Fourth Military Medical University Xi'an 710032 China; ^2^ State Key Laboratory of Transducer Technology Shanghai Institute of Microsystem and Information Technology Chinese Academy of Sciences Shanghai 200050 China; ^3^ Center of Materials Science and Optoelectronics Engineering University of Chinese Academy of Sciences Beijing 100049 China; ^4^ School of Physical Science and Technology ShanghaiTech University Shanghai 200031 China; ^5^ Institute of Brain‐Intelligence Technology Zhangjiang Laboratory Shanghai 200031 China; ^6^ Shanghai Research Center for Brain Science and Brain‐Inspired Intelligence Shanghai 200031 China

**Keywords:** diabetes mellitus, injectable silk gel scaffold, macrophage polarization, osseointegration promotion

## Abstract

As a chronic inflammatory disease, diabetes mellitus creates a proinflammatory microenvironment around implants, resulting in a high rate of implant loosening or failure in osteological therapies. In this study, macroporous silk gel scaffolds are injected at the bone–implant interface for in situ release of sitagliptin that can regulate macrophage response to create a prohealing microenvironment in diabetes mellitus disease. Notably, it is discovered that sitagliptin induces macrophage polarization to the M2 phenotype and alleviates the impaired behaviors of osteoblasts on titanium (Ti) implants under diabetic conditions in a dose‐dependent manner. The silk gel scaffolds loaded with sitagliptin elicite a stronger recruitment of M2 macrophages to the sites of Ti implants and a significant promotion of osteointegration, as compared to oral sitagliptin administration. The results suggest that injectable silk/sitagliptin gel scaffolds can be utilized to modulate the immune responses at the bone–implant interface, thus enhancing bone regeneration required for successful implantation of orthopedic and dental devices in diabetic patients.

## Graphical abstract

A silk gel scaffold loaded with sitagliptin is developed to in situ regulate macrophage polarization from M1 to M2 phenotypes and improve osteointegration of endosseous implants at the bone–implant interface under diabetes conditions. The study provides a new insight into the mechanisms underlying diabetes‐induced implant failure and represents an innovative strategy to promote osteointegration in diabetic patients with hyperglycaemia.

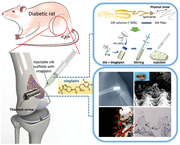

## 1 Introduction

Load‐bearing implants including Titanium (Ti) and its alloys have been extensively used in orthopedic and dental therapies.^[^ ^1^ ^]^ Osseointegration, which involves a cascade of biological processes that occur at the tissue‐implant interface and during the final formation of direct bone–implant contact, is essential for a successful implantation of orthopedic and dental devices.^[^ ^2^, ^3^ ^]^ However, failed integration of implants with bone can be caused by an acute foreign body response (FBR) to the exogenous immunogens or prolonged and chronic inflammatory at the bone–implant interface. Successful osseointegration, on the other hand, depends on a mild local inflammatory response, which promotes vascularization and new bone formation around the implants.^[^ ^4^ ^]^ These responses to implants are specifically directed by macrophages including the proinflammatory M1 macrophages and the alternatively activated prohealing M2 macrophages, which may change their phenotypes to modulate inflammatory or healing functions.^[^ ^5^ ^]^

Studies have shown that implant stability is usually jeopardized by delayed or impaired bone healing at the bone–implant interface in diabetic patients, with the underlying mechanism remaining unclear.^[^ ^6^ ^]^ Previous approaches that directly targeted osteoblast or osteoclast cells have resulted in very limited improvement in Ti osteointegration.^[^ ^7^, ^8^ ^]^ Recent studies support the view that macrophage transformation into the M2 phenotype is beneficial for bone formation.^[^ ^9^ ^]^ Research has been focused on strategies that alter the surface roughness and topography of Ti implants to modulate the inflammatory response, but these strategies have little meaningful effects in diabetes mellitus (DM) disease.^[^ ^10^, ^11^ ^]^ At the same time, treatment focused on targeting macrophages systemically is not ideal because it may impair the immune system as a whole.^[^ ^12^ ^]^ Although administration of specific oral antidiabetic drugs including sitagliptin, glimepiride, and insulin have been demonstrated to enhance new bone formation under DM conditions,^[^ ^13^, ^14^ ^]^ they may exhibit undesirable side effects including hepatic and gastrointestinal damage.^[^ ^15^ ^]^ The phenotypic switch from M1 to M2 does not readily occur at the bone–implant interface under diabetic conditions. Prolonged and uncontrolled M1‐mediated inflammation in DM disease conditions will create a proinflammatory microenvironment at the bone–implant interface and lead to a failure of Ti osteointegration.^[^ ^16^, ^17^ ^]^ Thus, methods for locally controlling the phenotypic conversion of macrophages could prevent the side effects of systemic anti‐inflammatory regimens while precisely modulating inflammatory responses at implant sites.

Sustained local drug release using injectable hydrogels permitting hydrogel formation to occur in situ upon delivery has recently attracted much attention.^[^ ^18^, ^19^, ^20^ ^]^ Although numerous injectable hydrogels have been developed for drug delivery and tissue repair, those prepared by solvent/additive‐free procedures and exhibiting macroporosity (crucial for cellular infiltration) and anti‐inflammatory properties remain challenging to fabricate. For example, the risks of chronic inflammation and foreign‐body reaction from the degradation products of synthetic biopolymers (such as lactic acid and carbon dioxide^[^ ^21^, ^22^, ^23^ ^]^) cannot be fully eliminated. Silk fibroin, a natural protein extracted from *Bombyx mori* silkworms with superior biocompatibility and adjustable biodegradability, has been extensively applied in biomedical engineering applications.^[^ ^24^, ^25^, ^26^ ^]^ In addition, the ability of silk fibroin to locally release cytokines has been demonstrated by an experiment in which fibroin‐mediated local release of cytokines induced macrophage differentiation without affecting the immune system; this suggests their use in the treatment of macrophage‐associated diseases.^[^ ^27^ ^]^

**Figure 1:**
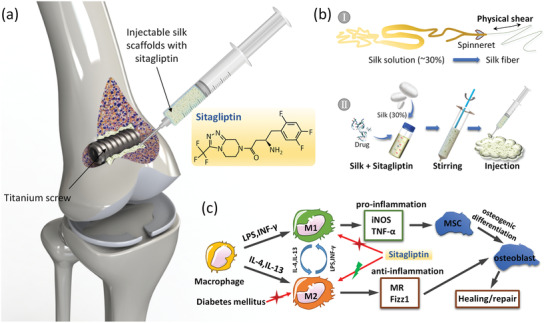
Regulation of macrophage phenotypic conversion at the bone–implant interface to promote osseointegration using injectable silk/sitagliptin gel scaffolds. a) Injectable silk gel scaffolds with sitagliptin at the bone–implant interface. b) The all‐aqueous process of injectable silk/sitagliptin gel scaffolds mimicking silk fiber spinning: I silk fiber spinning in silkworm and II fabrication of silk gel scaffolds. c) The effect of sitagliptin on the conversion of M1 to M2 macrophage phenotypes and osteogenesis under diabetic conditions.

In this study, injectable macroporous silk gel scaffolds loaded with sitagliptin were fabricated by an all‐aqueous process and injected into the bone–implant interface to locally release sitagliptin. This procedure modulated macrophage responses to create a prohealing microenvironment under DM conditions (**Figure** **1**a,b). Notably, we demonstrated for the first time that sitagliptin‐induced macrophage conversion into the M2 phenotype and alleviated impaired behavior of osteoblasts on Ti under DM conditions in a dose‐dependent manner. In vivo results show that the silk/sitagliptin gel scaffolds induced effective recruitment of M2 macrophages to the sites of Ti implants and more successful osseointegration compared to oral sitagliptin administration. These results represent a promising strategy to enhance bone regeneration and reverse delayed bone healing after orthopedic and dental therapies for diabetic patients.

## 2 Results and Discussion

### 2.1 Macrophage Phenotypic Conversion and Osteoblast Regulation Using Sitagliptin Under Diabetic Conditions

The M1 and M2 macrophage phenotypes play an important role in regulating the osteogenic functions of bone marrow stromal cells (MSC) and osteoblasts, which are essential for bone regeneration (Figure 1c).^[^ ^28^ ^]^ A timely and smooth shift from proinflammatory M1 into anti‐inflammatory M2 macrophages is essential for bone regeneration. However, M1 macrophages fail to convert into the M2 phenotype in DM disease conditions,^[^ ^9^, ^29^ ^]^ resulting in an increased production of proinflammatory cytokines, which suppress osteogenesis.

**Figure 2:**
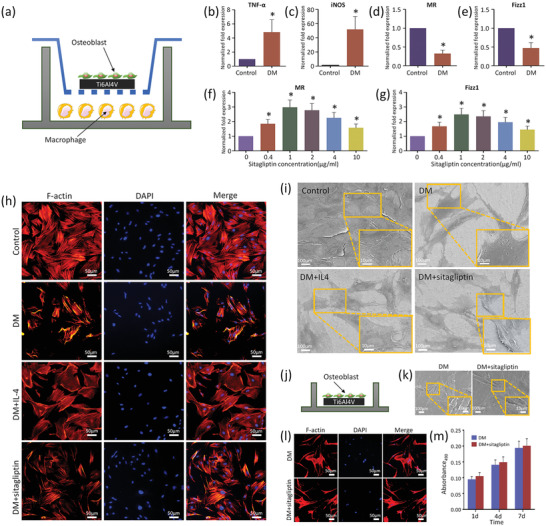
Sitagliptin attenuates the diabetes (DM)‐induced inhibition of the biological functions of osteoblasts by converting macrophage phenotypes from M1 to M2. a) Schematic diagram shows the co‐culture of macrophages and osteoblasts on Ti implants in a transwell system. b‐e) Enhanced the expression of M1 markers TNF‐*α* and iNOS, but suppressed the expression of M2 markers MR and Fizz1 in DM macrophages compared to control macrophages. f, g) Conversion of M1 to M2 phenotypic DM macrophages by sitagliptin in a dose‐dependent manner after 24 hours as shown by reduced expression of MR and Fizz1 markers after 24 h. h) Confocal laser scanning microscopy images of DM osteoblast F‐actin distribution and nuclei in different groups after 3 day co‐culture showing effect of IL‐4 and sitagliptin treatment (red, Rhodamine‐phalloidin for F‐actin; blue, DAPI for nucleus). i) Representative SEM images of osteoblast on Ti surface after incubation for 7 days. j) Schematic illustration of direct culture of osteoblast. k) Representative SEM images of osteoblast on Ti surface after incubation for 7 days. l) Confocal laser scanning microscopy images of DM osteoblast F‐actin distribution and nuclei in DM and DM+sitagliptin groups. m) Assays for cell proliferation in DM and DM+sitagliptin groups after 1, 4, and 7 days of incubation. *p < 0.05 versus control group.

Sitagliptin (Figure 1a), recognized as a DPP‐4 inhibitor to improve the blood glucose level and insulin resistance situation in diabetic patients, has been reported to reduce bone loss and increase bone strength in diabetic rats.^[^ ^30^ ^]^ Although some studies have demonstrated that sitagliptin shows some anti‐inflammatory effects,^[^ ^31^ ^]^ the action mechanism of sitagliptin on osseointegration promotion still remains unclear. In this study, coculture of macrophages and osteoblasts on Ti implants in a transwell system was carried out to investigate the effect of sitagliptin on osseointegration (**Figure** **2**a). DM caused an increase in the production of proinflammatory cytokines, such as iNOS and TNF‐*α*, and a decrease in the production of anti‐inflammatory cytokines such as MR and Fizz1 (Figure 2b–e). The effect of DM on macrophage polarization was also evaluated by ELISA analysis and immunofluorescence staining (Figure S1, Supporting Information). The amounts of TNF‐a and IL‐6 secreted by macrophages in DM group were higher than those secreted by macrophages in the control group, while the amounts of IL‐10 were lower. CD68 (a pan‐macrophage marker), iNOS (an M1 macrophage marker), and CD206 (an M2 macrophage marker) were selected to characterize macrophage phenotype. The semiquantitative analysis of iNOS and CD206 was consistent with the results of RT‐qPCR and ELISA analysis. Sitagliptin induced a biphasic effect on the expression of M2 markers with an optimal concentration of ≈1 µg mL^−1^, and inhibited inflammation by converting to the M2 macrophage phenotype (Figure 2f,g). Sitagliptin treatment can reduce plasma levels of proinflammatory markers in diabetic patients and inhibit the inflammatory response through suppression of NF‐*κ*B transcriptional activity.^[^ ^32^, ^33^ ^]^ Thus, an optimal drug dosage would suppress active inflammatory pathways and guide local host responses toward desirable phenotypes.^[^ ^34^ ^]^ Treatment with 1 µg mL^−1^ sitagliptin significantly alleviated the impaired behavior of osteoblasts, as compared with the untreated diabetic group, evidenced by better cell adhesion, morphology, extracellular matrix (ECM) mineralization, osteogenic differentiation, and proliferation (Figure 2h,i; and Figures S2–S5, Supporting Information).

Clinical studies show that the presence of a greater proportion of M1 phenotype macrophages is highly correlated with the failure of artificial joints.^[^ ^35^ ^]^ Since the IL4 cytokine is effective in converting M1 to M2 macrophage phenotypes, IL‐4 administration should be important for DM‐associated bone regeneration. Coculturing of osteoblasts with IL‐4 treated macrophages in transwells markedly alleviated the impairment of osteoblast (Figure 2h,i; and Figures S2–S5, Supporting Information), suggesting that transforming macrophages to an anti‐inflammatory M2 phenotype could enhance bone regeneration in DM disease.

To eliminate the direct effect of sitagliptin on osteoblasts, we further investigated the influence of DM+sitagliptin on the behaviors of osteoblasts at the same concentration (1 µg mL^−1^). Sitagliptin treatment did not obviously improve cell proliferation, ECM mineralization, production of alkaline phosphatase, or expression of osteogenic genes on Ti as compared to diabetic conditions by direct culture (Figure 2j–m; and Figure S6, Supporting Information). The results suggest that sitagliptin inhibits impaired osteoblast behavior on Ti under DM conditions by inducing macrophages conversion into the M2 phenotype.

### 2.2 In Vitro Sitagliptin Release by Silk Gel Scaffolds to Regulate Macrophage Phenotypic Conversion

**Figure 3:**
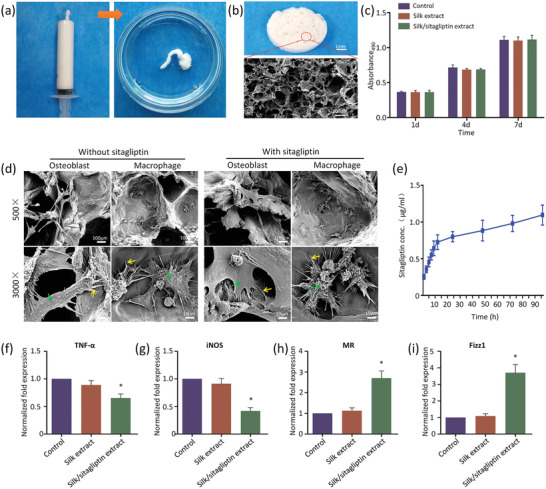
Sitagliptin release of the silk gel scaffolds to regulating macrophage polarization in vitro. a) Injectability of the silk gel scaffold in deionized water. b) Photograph (upper) and SEM images (lower) of silk gel scaffolds. c) Results of assays for L929 mouse cell proliferation after 1, 4, and 7 days. d) SEM images showing attachment behavior of osteoblast and macrophage on silk gel scaffolds. Green arrows label the osteoblast or macrophage. Yellow arrows label the filopodia. e) The released concentration of sitagliptin from silk gel scaffolds over 96 h. f–i) The expression of M1 markers TNF‐*α* and iNOS and M2 markers MR and Fizz1 for control macrophages compared to those incubated with silk and silk/sitagliptin scaffold extract after 24 h culture. *p < 0.05 versus control group.

Our previous study showed reduced inflammatory cytokines in cultured human umbilical vein endothelial cells (HUVECs) in vitro, and promoted angiogenesis in vivo after administrated of sitagliptin orally.^[^ ^36^ ^]^ However, the local effective concentration (1 µg mL^−1^) can only be achieved at a large dose of oral drug administration (about 4‐time higher than normal). A large dose of oral sitagliptin administration could cause nephrotoxicity according to American Diabetes Association standards.^[^ ^37^, ^38^ ^]^ Using an in situ drug release system can significantly decrease adverse side effects compared with systemic treatment which is effective only when sufficient doses are given to achieve the desired concentration at the Ti‐bone interface. Injectable porous silk hydrogels loaded with sitagliptin were prepared via an all‐aqueous process by mimicking the liquid crystalline spinning process of silk fibers using 30% silk solution (Figures 1b and **3**a). By trapping gas bubbles in the silk solution during the gelation process, a tunable porous structure was generated, and a wide range of mechanical properties of the silk gel scaffolds could be adjusted by different gelation times, which performed better macroporosity and lower brittleness compared with scaffolds prepared by traditional methods according to the previous study.^[^ ^39^ ^]^ This optimized scaffold is expected to show superior performance and flexible application for bone regeneration. The silk gel scaffolds are promising as injectable drug encapsulation matrices because they can rapidly solidify (< 10 min) and be facilely loaded with drugs. As shown Figure 3b, the silk gel scaffolds (wet state) showed a favorable macroporous structure, which constructed a structure of trabeculae to affect the osteoconductive properties of scaffold materials and the resultant bone tissue ingrowth and vascularization.^[^ ^40^ ^]^ Silk gel scaffolds will gradually lose their structural integrity and are filled by invading cells as the silk gel scaffolds degraded (Figure S11b, Supporting Information). The results from a biocompatibility assay show that mouse L929 cells grew vigorously in an extract of the silk/sitagliptin scaffolds (Figure 3c; and Figure S7, Supporting Information). Osteoblasts and macrophages exhibited typical morphology and had an extensive interaction with the porous silk gel scaffolds (Figure 3d).

Since sitagliptin inhibited inflammation and promote bone regeneration in a dose‐dependent manner (Figure 2f,g), the optimal release pattern of sitagliptin from the silk/sitagliptin gel scaffolds was manipulated by controlling the drug concentration in the silk and the processing parameters of silk gel scaffolds (see the Experimental Section) (Figure 3e). Drug release behavior of the silk gel scaffolds was studied in vitro using the liquid chromatography‐mass spectrometry system (LC‐MS) approach. The results indicate that sitagliptin release was rapid during the first 10 h and then gradually slowed down afterward, approaching the optimum concentration of ≈1 µg mL^−1^. Representative high performance liquid chromatography (HPLC) chromatograms of sitagliptin released from the silk/sitagliptin group after 1 and 96 h are shown in Figure S8 (Supporting Information). Only very small portion (<2%) of the drug was released to the surrounded matrix by diffusion in 4 days, probably due to the typical high concentration of silk fibroin and thus the dense molecular networks that prevent the drug from spreading out of the substance. The drug encapsulated in the interior (>98%) will offer a continuous drug release as the silk gel scaffolds degraded in vivo. In addition, silk materials have been testified to preserve the functionality of the cytokines that are loaded in silk.^[^ ^41^, ^42^ ^]^ Thus, sitagliptin‐loaded silk gel scaffolds will have a sustained effect on the Ti‐bone interface after implantation. Nevertheless, an ideal model in vitro that could simulate silk biodegradation in vivo has not been fully constructed. In future experiments, the in vitro drug‐release detection method of drug‐loaded silk gel scaffolds is worthy of further exploration.

To test the regulatory effect of the silk gel scaffolds on macrophage phenotypic conversion, the macrophages were cultured in medium with and without silk scaffold extract for 24 h. We then performed qPCR to analyze the gene expression of M1 phenotype markers (TNF‐*α* and iNOS) and M2 phenotype markers (Fizz1 and MR). There was no significant difference in expression of TNF‐*α*, iNOS, Fizz1, and MR between the blank control and silk extract group, while the silk/sitagliptin group showed a significant conversion to the M2 phenotype (Figure 3f–i). ELISA analysis was conducted to further confirm the anti‐inflammatory switch. The results showed the levels of the cytokines TNF‐a, IL‐6, and IL‐10 secreted by macrophages in silk extract group were comparative to that in control group. The amounts of TNF‐a and IL‐6 in silk/sitagliptin extract group were lower than those in both the control and silk extract group, whereas the amounts of IL‐10 were higher (Figure S9, Supporting Information). The effect of silk gel on macrophage phenotype and bone formation in vivo (Figure S10, Supporting Information) was also evaluated. Both the DM and DM+silk group exhibited similar inflammatory response in which expressions of the iNOS‐positive cells and CD163‐positive cells were comparable in the DM + silk group than those in the DM group. The V–G staining results showed that new bone formation around implants was almost the same in the DM + silk group with that in the DM group at 4 weeks.

### 2.3 Silk Degradation and Bone Regeneration Around Ti Implants

**Figure 4:**
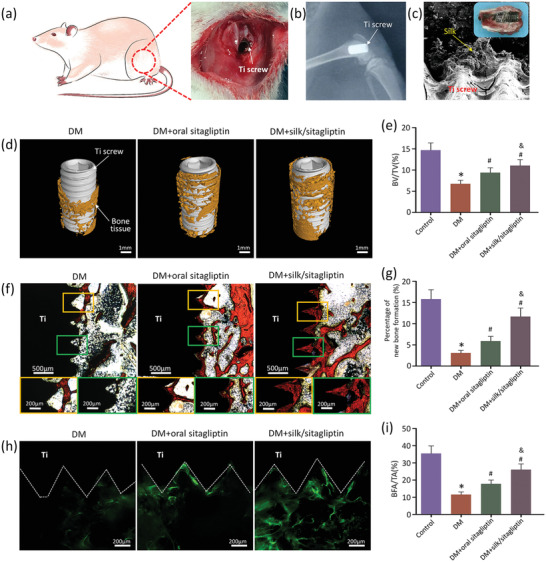
New bone formation at the bone–implant interface after 4 weeks. a) Ti implants were placed in femur defects which were preinjected with silk gel scaffolds. b) A representative radiograph of a femur with implants. c) SEM micrograph of silk gel scaffolds around the implant; inset: cross‐sectional view. d) 3D‐reconstructed images from micro‐CT analysis show the implant and the bone formation in the region of interest (ROI). e) The ratio of bone volume to total volume (BV/TV) in the ROI around implants. f) Histological images of new bone formation around the implants by Van Gieson staining for newly formed bone (red). g) Histomorphometric measurement of new bone formation around the implants. h) Fluorescent images of histological sections showing new mineral deposition, labeled by calcein (green), proximate to the threads of the Ti screw implant (dotted line). i) The ratio of bone formation area to the total area of ROI (BFA/TA) in fluorochrome analysis. *p < 0.05 versus control group (healthy rats); #*p* < 0.05 versus DM group; &*p* < 0.05 versus DM + oral sitagliptin group.

To examine the distribution of silk gel scaffolds around the implant and new bone formation, silk/sitagliptin gel scaffolds were injected into the defects in the femurs of rats. Then, screws made of Ti alloy were implanted in these defects (**Figure** **4**a,b). Typical sectional images of samples show the silk gel scaffolds on the surface of the implant and at the Ti‐bone interface (Figure 4c; and Figure S11a, Supporting Information). The silk gel scaffolds displayed favorable degradability over 30 days (Figure S11b, Supporting Information) and thus a promising strategy of sustained drug release to regulate macrophage phenotypic conversion and bone regeneration. In vitro degradation of silk gel scaffolds was also investigated using phosphate‐buffered saline (PBS) (control, pH 7.4), simulated body fluid (SBF) and SBF + proteinase K^[^ ^25^ ^]^ (Figure S12, Supporting Information). The silk gel scaffolds in PBS (control) and SBF exhibited minimal degradation (< 10%) over 14 days, while a maximum degradation was observed in the SBF + proteinase K group (≈85%) (Figure S12b, Supporting Information). The degradation was confirmed by scanning electron microscope (SEM) images of the degraded silk gel scaffolds (Figure S12a, Supporting Information).

To evaluate the new bone formation and osseointegration at the Ti‐bone interface, micro‐CT and histological analysis was performed 4 weeks after surgery. Micro‐CT analysis showed that the DM group had much less bone tissue around the implants observed compared with the control group (healthy rats), indicating that DM compromises the osteointegration of Ti implants. Meanwhile, oral sitagliptin treatment only slightly improved bone regeneration around the Ti implants. By contrast, in situ release of sitagliptin at the Ti‐bone interface in the silk/sitagliptin group significantly improved new bone formation around implants, as evidenced by the increased BV/TV (bone volume out of total volume) and trabecular structural features of the new bone (Figure 4d,e; and Figure S13, Supporting Information). Similar results were also be observed in Van Gieson stained images of Ti‐bone interface (Figure 4f,g; and Figure S14, Supporting Information). New bone mineralization (labeled by fluochrome) around implants was also observed (Figure 4h,i; and Figure S15, Supporting Information). The ratio of bone formation area to the total area (BFA/TA) (calculated from the fluorochrome label) in the DM group was notably lower than that in the silk/sitagliptin group. These results suggest that in situ sitagliptin release at the Ti‐bone interface using silk gel scaffolds significantly improves new bone formation surrounding Ti implants.

### 2.4 Regulation of Macrophage Phenotypic Conversion to Improve Osseointegration at the Bone–Implant Interface

**Figure 5:**
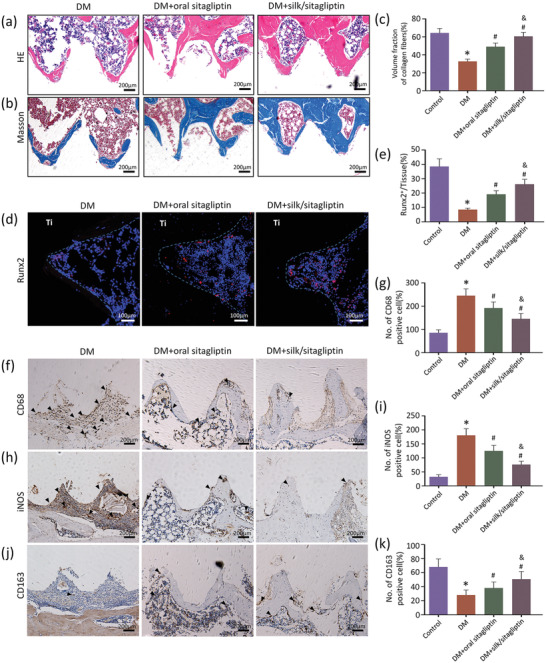
Histological analysis of bone formation and macrophage phenotypic conversion surrounding in vivo Ti implants. a) Representative HE images of implants harvested at 4 weeks showing inflammatory cells infiltration. b) Representative Masson's trichrome staining images showing the expression of collagen (blue). c) The analysis of the proportion of collagen volume in the ROI around implants. d) Expression levels of the osteogenic marker, Runx2, labeled by an osteoblast marker around Ti implants. e) Results of semiquantitative results of Runx2 positive area/tissue area (Runx2^+^/Tissue). Immunohistochemical images of f) CD68, h) iNOS and j) CD163 in the tissue around the implant. The numbers of cells positive for g) CD68, i) iNOS and k) CD163 in the ROI were counted. *p < 0.05 versus control group (healthy rats); #*p* < 0.05 versus DM group; &*p* < 0.05 versus DM + oral sitagliptin group.

The osteogenesis enhancement by silk/sitagliptin gel scaffolds in diabetic rats could be attributed to their promotion of an anti‐inflammatory response at the bone–implant interface. An obvious inflammatory response in diabetic rats is observed from the haematoxylin and eosin (HE) staining results, while administration of oral sitagliptin slightly inhibited inflammatory cell infiltration. In situ sitagliptin release using injected silk gel scaffolds at the Ti‐bone interface resulted in decreased numbers of inflammatory cells (**Figure** **5**a; and Figure S16, Supporting Information). New collagen synthesis, an indicator for new bone formation, also increased in the silk/sitagliptin group in comparison with the DM and oral sitagliptin groups (Figure 5b,c; and Figure S16, Supporting Information). In addition, the expression of the osteogenic transcription factor Runx2 and osteogenic marker osteocalcin (OCN) (indicators of cell commitment to an osteogenic lineage) in the silk/sitagliptin group was greater than that in the DM and oral sitagliptin groups (Figure 5d,e; and Figure S17, Supporting Information).

Since diabetes is associated with chronic activation of M1 macrophages,^[^ ^43^ ^]^ we observed how sitagliptin impacts inflammatory cell infiltration and the phenotypic conversion of macrophages around Ti implants using immunohistochemical staining. Inflammatory cell infiltration was determined by CD68; and M1 to M2 phenotype conversion of macrophages was determined by iNOS and CD163 expression, respectively (Figure 5f–j; and Figure S18, Supporting Information). Our results were corroborated by the quantitative immunohistochemistry data (Figure 5g–k). Diabetes caused a significant decrease in the expression of the M2 marker CD163, an increase in CD68 and an increase in the M1 marker iNOS; this indicated both an increased number of infiltrated macrophages and decreased M1 to M2 phenotypic conversion. In situ release of sitagliptin at Ti‐bone interface using silk gel scaffolds resulted in much higher numbers of CD163 (M2 marker)‐positive cells than the DM group, while the numbers of iNOS (M1 marker) and CD68‐positive cells were diminished. By contrast, these trends were much attenuated when sitagliptin was administered orally. These studies demonstrate the potential application of silk gel scaffolds to modulate macrophage phenotypic conversion in order to enhance bone regeneration required for successful implantation in patients with chronic inflammatory diseases. Considering that macrophages mediate the initial inflammatory response and participate in host response during the whole bone healing process, evaluation of the macrophage phenotype changes throughout the osseointegration stage needs to be done in the future.

## 3 Conclusion

We report an innovative technique for in situ regulation of macrophage polarization at the bone–implant interface to improve osseointegration under diabetic conditions using injectable silk/sitagliptin gel scaffolds. In order to create a prohealing microenvironment in DM disease, in situ and sustained release of sitagliptin was achieved with injectable macroporous silk/sitagliptin gel scaffolds formed in an all‐aqueous process. The controlled release of sitagliptin at the bone–implant interface promotes macrophage transformation from M1 to M2 phenotypes and osteointegration of Ti implants under diabetic conditions. Our findings provide a new insight into the mechanisms underlying diabetic‐induced implant failure, and represent new strategies to enhance the osteointegration of endosseous implants, spine, or trauma screw and cementless joint prostheses, and reduce implant failure in diabetic patients with hyperglycemia.

## 4 Experimental Section

##### Preparation of the Ti6Al4V Specimen

A Ti6Al4V alloy was machined into screws (3 mm in diameter, 6 mm in length) and circular discs (10 mm in diameter and 1 mm in thickness) at the Northwest Institute for Nonferrous Metal Research, Xi'an, China. Screws were used in the in vivo experiments and circular discs were used in the in vitro experiments.

##### Preparation of Silk Fibroin Aqueous Solutions

The regenerated silk fibroin aqueous solution was prepared as follows: 10 g silkworm cocoons from *B. mori* were cut into small pieces and degummed by boiling in 0.02 m Na_2_CO_3_ (Sigma‐Aldrich, USA) solution (4 L) for 30 min. After boiling, the degummed silk fibres were washed for 3 × 20 min in distilled water and dried for more than 12 h at room temperature (RT). Then, the dried silk fibres were dissolved in 40 mL 9.3 m LiBr solution (Sigma‐Aldrich, USA) at 60 °C for 4 h. The blended solution was then dialysed (MWCO 3.5 kDa, Pierce, USA) in deionized water for 48 h and centrifuged for 2 × 20 min at 12 000 r.p.m to obtain the purified silk solution in water (concentration ≈8%). To obtain the highly concentrated silk, the aqueous silk (concentration ≈8%) was injected into a dialysis bag (MWCO 3.5 kDa, Pierce, USA) and subjected to airflow at 4 °C to evaporate the water until a concentration of ≈30% was reached.

##### Preparation of Silk/Sitagliptin Gel Scaffolds

A mixed solution of silk fibroin and sitagliptin was prepared by adding sitagliptin (407.32 g mol^−1^) to a concentrated silk fibroin (≈30%). Briefly, to obtain the sitagliptin‐loaded silk fibroin aqueous solution, 2.5 mg sitagliptin was added into 1 mL silk fibroin aqueous solution. A glass rod was used to stir the concentrated silk fibroin solution in the syringe (20 mL type) for 2 min at a stirring speed of ≈100 r min^−1^ (mimicking spinning speeds of silk fiber of 10–20 mm s^−1^) by hand.^[^ ^39^ ^]^ For in vivo experiment, the foaming silk gel was directly injected into animals. For in vitro experiments, the foaming silk gel was placed at RT to form silk/sitagliptin scaffolds, which were then cut into discs. Small silk scaffold discs with diameters of 10 mm and heights of 3 mm (containing ≈0.25 mg sitagliptin) were prepared and sterilized through 60Co *γ*‐irradiation at a dose of 25 kGy.

##### In Vitro Release Profile of Sitagliptin

The concentrations of released sitagliptin from the silk gel scaffolds were determined by LC‐MS (Shimadzu, Kyoto, Japan). Briefly, silk scaffold samples were transferred into a centrifuge tube and immersed in deionized water (2 mL per sample) and incubated at 37 °C. At predetermined time intervals (1, 3, 5, 7, 9, 12, 24, 48, 72, and 96 h), 0.5 mL supernatant was removed from each sample and additional 0.5 mL water was added. The removed supernatant was collected and subjected to LC‐MS analysis for sitagliptin quantitation.

##### The Isolation and Culture of Osteoblasts and Macrophages

Primary rat osteoblasts were isolated from the calvarias of neonatal Sprague‐Dawley (SD) rats using the enzymatic isolation method. Experiments were conducted according to an animal experimental protocol approved by the Fourth Military Medical University. Briefly, the calvaria tissues were extracted and minced into 1 × 1 mm^2^ fragments. Then, these fragments were digested with 0.5% trypsin at 37 °C for 15 min. After three washes with PBS, the bone fragments were transferred to a culture flask, cultured in Dulbecco's modified Eagle's medium supplemented with 10% fetal bovine serum (FBS) and 1% penicillin/streptomycin, and then incubated in a humidified atmosphere of 5% CO_2_ at 37 °C. Osteoblasts between the second and the third passages were used as seeds for coculture.

To acquire macrophages, bone marrow from adult rats was flushed from femurs and tibias and then plated in RPMI 1640 medium supplemented with 10% FBS, 1% penicillin/streptomycin, and 20 ng mL^−1^ rat macrophage colony‐stimulating factor (Pepro Tech, Inc.). Cells were seeded in 75 cm^2^ culture flasks and then incubated in a humidified atmosphere of 5% CO_2_ at 37 °C for 7 days. The completed culture media containing 25 mmol L^−1^ glucose (high glucose) and 500 µmol L^−1^ BSA‐conjugated palmitate (high fat) was used as the mimic milieu of type 2 diabetes.^[^ ^44^ ^]^

##### In Vitro Degradation of Silk Gel Scaffolds

The in vitro degradation assays were carried out in PBS (control, pH 7.4), simulated body fluid (SBF, Sigma‐Aldrich, USA) and SBF + proteinase K at 37 °C in a 12‐well plate with 0.05% sodium azide. Samples were replenished with SBF + proteinase K solution every 72 h. On days 1, 3, 7, and 14, silk gel scaffolds were washed with deionized water, freeze dried, and weighed. Both initial and degraded silk gel scaffolds were weighed using an electronic balance (Sartorious, AG Germany). The morphology of the silk gel scaffolds was examined using a SEM

(1) $$\begin{array}{ccc} & & \mathrm{Percent}\;\mathrm{mass}\;\mathrm{remaining}=(Mt/Mi)\times 100\%\\ & & Mt\;\mathrm{is}\;\mathrm{the}\;\mathrm{weight}\;\mathrm{on}\;\mathrm{the}\;\mathrm{specified}\;\mathrm{day},\;\mathrm{and}Mi\;\mathrm{is}\;\mathrm{the}\;\mathrm{initial}\;\mathrm{weight}. \end{array}$$

##### In Vitro Distribution Test

A 3 mm diameter injury was generated in bilateral femoral condyle of rats in a perpendicular orientation. The silk gel scaffolds of ≈0.05 mL prepared as described above were injected into the defects of the femoral heads of which the volume is 0.042 mL (calculated according to the drill size), and then titanium screws were inserted into the femoral condyle defects that had been filled with the gel scaffolds. The silk gel scaffolds can be squeezed into the bone trabecular to realize the slow release of sitagliptin around the implants. For in vitro study, each distal femur was cut along the edge of the nail, and the bone tissue surrounding the implant was carefully divided into two parts and forced apart from the implant surface, with minimized the damage on the interface tissues. Then, the distribution of the silk gel scaffolds, which partly covered the surface of the nail and bone–implant interface, was assessed by a dissection microscope and an SEM. For in vivo study, on days 1, 10, 20, and 30 after implantation, rats were sacrificed, and specimens were obtained for Van‐Gieson staining. For detailed procedures, see “Histomorphometric analysis of bone healing around implants” in the Experimental Section.

##### Transwell

Osteoblasts were cocultured with macrophages using 24‐Transwell plates with a 0.4 µm pore size (Corning Life Sciences). Macrophages were seeded on the bottom chamber of the transwell, and osteoblasts were seeded on the top chamber.

##### Cell Viability Assessment by MTT Assays

Osteoblast proliferation was assessed using the methylthiazol tetrazolium (MTT) assay at 1, 4, and 7 days of incubation. Additionally, the L929 fibroblast cell line was used for cytotoxicity assessment with silk extract in the same way. Briefly, 100 µL MTT (0.5 mg mL^−1^) was added, and the cells were incubated for 4 h at 37 °C for formazan formation. Then, dimethyl sulfoxide was added to dissolve the formazan crystals, and optical density values were measured using a spectrophotometer at 490 nm.

##### Cell Adhesion and Morphology Examination

To evaluate osteoblast adhesion, after incubation for 3 days, cellular actin filaments and nuclei were visualized by staining with rhodamine‐phalloidin (Molecular Probes, Eugene, OR) for 20 min and 40, 60‐diamidino‐2‐phenylindole (DAPI, Sigma, St. Louis, MO) for 15 min. Cells were viewed with a confocal scanning laser microscope (Olympus Fluoview), and digital images were captured in the TIFF format using the Olympus Fluoview software. The cell area and cell density were measured using the ImageJ software package. Five different substrate fields were measured per sample, and three separate samples were measured in each group. Cell morphology was observed in each group after 7 days of incubation using an SEM (S‐4800, Hitachi, Japan) operating at 15 kV and a semiautomatic interactive image analyzer. Before observation, the samples were fixed in 2% v/v glutaraldehyde at 4 °C overnight. After dehydration through an ethanol series and critical point drying, the samples were sputtered with gold for SEM observation.

##### Osteogenic Differentiation and Mineralization Assay

After 7 days of induction, alkaline phosphatase (ALP) staining was performed using the 5‐Bromo‐4‐Chloro‐3‐Indolyl Phosphate/p‐Nitro‐Blue tetrazolium (BCIP/NBT) alkaline phosphatase color development kit (Beyotime Co., Shanghai, China) for 30 min. The ALP staining results were observed under an optical microscope. The ECM mineralization of samples was evaluated with Alizarin Red staining (Sigma, St. Louis, MO) after 21 days of incubation. After being washed three times with PBS and fixed in 75% ethanol for 1 h, the samples were stained with 40 × 10^−3^ m Alizarin Red in distilled water (pH = 4.2) for 20 min at RT. The Alizarin Red solution was removed by washing twice with distilled water. The stained cells were observed under a light microscope. For quantitative analysis, the stain was dissolved with 10% cetylpyridinium chloride in 10 × 10^−3^ m sodium phosphate (pH = 7.0), and the absorbance was measured at 630 nm.

##### Quantitative Real‐Time PCR

To quantify the levels of gene expression, total RNA was extracted from osteoblasts and macrophages and converted to cDNA using the PrimeScriptRT reagent kit (Takara). Gene expression was quantitated by qPCR using SYBR Premix ExTaq II (TaKaRa) on the CFX96PCR System (Bio‐Rad). The primers used in this study are listed in Table S1 (Supporting Information), and *β*‐actin was used as a housekeeping gene.

##### Immunofluorescence Staining

To analyze macrophage polarization, the samples were fixed in 4% paraformaldehyde for 30 min, then permeabilized with 0.1% Triton X‐100 at room temperature. After incubated for 1 h in PBS containing 20% goat serum and 3% bovine serum albumin at room temperature to block nonspecific adsorption, samples were incubated with mouse monoclonal anti‐CD68 (Abcam, Cambridge, MA) and rabbit polyclonal anti‐CD206 (Abcam) primary antibodies to identify M2 macrophages and with mouse monoclonal anti‐CD68 (Abcam) and rabbit polyclonal anti‐iNOS (Abcam) primary antibodies to identify M1 macrophages. Next, the samples were incubated with Alexa Fluor 488 (green) antirabbit IgG (Abcam) and Alexa Fluor 647 (red) antimouse IgG (Abcam) secondary antibodies. Finally, the samples were observed under a confocal laser scanning microscope (TCS SP5, Leica, Buffalo, NY).

##### ELISA Assay

After 24 h of culture, medium of macrophages was harvested. The secretion of TNF‐a, IL‐6, and IL‐10 was detected by using the enzyme‐linked immunosorbent assay (ELISA). The concentrations were detected using ELISA kits (MultiSciences Biotech Co. Ltd, Hangzhou, Zhejiang, China), according to the manufacturer's instructions.

##### Preparation of the High‐Fat Diet (HFD)/Streptozotocin (STZ)‐Induced Diabetic Rats

Male SD rats (200–250 g) were purchased from the Experimental Animal Breeding Centre, the Fourth Military Medical University (Xi'an, Shaanxi, China). All rats were housed in an air‐conditioned room at 22 ± 1 °C with a humidity of 50 ± 10% and a constant 12 h light and 12 h dark cycle. The rats were fed and provided tap water ad libitum. All animal experiments were overseen and approved by the Animal Care and Use Committee of the institute before and during the experiments.

To induce diabetes, rats were fed with a HFD (60% fat; Research Diets, D12492) for 4 weeks. After 4 weeks, the HFD‐fed rats were injected intraperitoneally with STZ dissolved in citrate buffer (pH 4.5) at a dose of 30 mg kg^−1^ body weight (b.w.), and the HFD was continued for an additional week. One week after the injection, the blood glucose level was measured by using a portable glucometer with blood collected from the tail vein. Animals with nonfasting blood glucose levels >300 mg dl^−1^ were considered diabetic.

##### Animal Surgery and Treatments

Six normal SD rats without diabetes were chosen as the control group. Eighteen diabetic rats were randomly divided into three groups: the diabetic group (*n* = 6), the diabetic + oral sitagliptin group (*n* = 6), and the diabetic + silk/sitagliptin group (*n* = 6).

Animals were anaesthetized with intraperitoneal injections of 10% chloral hydrate (3.3 mL kg^−1^). After anaesthesia, the right hind limb was prepared by shaving and cleaning using ethanol and chlorhexidine. A 10 mm incision was made by using a scalpel at the medial side of the knee joint, and the extensor of the knee joint was dislocated laterally. With the knee in flexion, an implant bed was made through each intercondylar notch with a rotary drill into the medullary canal of the femur via the distal femoral metaphysis, and an implant was placed into the femur below the articular surface. The silk/sitagliptin group received injection of silk foam loaded with sitagliptin into the medullary. Then, the extensor mechanism was reconstructed, soft tissues were sutured in separate layers and all the animals received intramuscular antibiotic injection for three postoperative days. The oral sitagliptin group was give sitagliptin (10 mg mL^−1^), dissolved in saline, by gavage (10 mg kg^−1^ day^−1^) according to previous studies^[^ ^45^ ^]^ and the clinical guidelines. The Control group and DM group were given an equivalent volume of saline, and all rats were treated for 4 weeks.

Animals were allowed free movement without any restriction. Four weeks after implantation surgery, the animals were sacrificed, and the femurs with implants were collected for the following evaluation. The animal experiments were carried out strictly in accordance with the National Institutes of Health Guidelines for the Use of Laboratory Animals and approved by the Fourth Military Medical University Committee on Animal Care (Animal Ethical Committee approval number: FMMU‐AEEA‐20180107).

##### Fluorochrome Labeling of New Bone Mineralization

After surgery, the animals were injected subcutaneously with calcein (10 mg kg^−1^, Sigma) to mark the real‐time process of osteogenesis at 1 week and 3 weeks. At 4 weeks, rats (*n* = 6 per group) were sacrificed, and femur specimens were obtained. After fixation in 10% formalin solution for 4 days, the specimens were dehydrated in a graded series of ethanol (70–100%) and embedded in methylmethacrylate (Sigma) solution that was polymerized at 37 °C within 7 days. Using an interlocked diamond saw (Leica Microtome, Wetzlar, Germany), thin sections (50 µm in thickness) were prepared. The sections were observed with a fluorescence microscope (Olympus), and images were acquired and analyzed by ImageJ software to determine the ratio of BFA/TA in the region of interest (ROI). For quantitative analysis, three slices of each sample were analyzed, with six samples in each group.

##### Micro‐CT Evaluation

After 4 weeks of implantation, the animals were sacrificed, the femurs were extracted (*n* = 6 per group) and fixed in 10% neutral buffered formalin for 4 days. Then, they were scanned in a micro‐CT system (Y.Cheetah X‐ray, Y.XLON, Germany) and reconstructed with an isotropic voxel size of 10 µm. A multilevel threshold procedure was applied to discriminate bone from other tissues. The ROI included a radius 100 µm from the implant surface. After 3D reconstruction, bone volume out of total volume (BV/TV) was calculated using Inveon Acquisition Workplace.

##### Histomorphometric Analysis of Bone Healing Around Implants

After micro‐CT scanning, the specimens were dehydrated in a graded ethanol series (70–100%) and then embedded in a methylmethacrylate solution that was polymerized at 37 °C within 1 week. Afterward, thin sections (≈50 µm in thickness) were prepared using the modified interlocked diamond saw (Leica Microtome, Wetzlar, Germany) and stained with 1.2% trinitrophenol and 1% acid fuchsin (Van‐Gieson staining). Bone formation was qualitatively measured with a standard light microscope (Leica) equipped with a digital image analysis system (Image‐Pro Plus software, Media Cybernetics, Silver Spring, USA). Prior to histomorphometric analysis, bone and material were pseudocoloured using Adobe Photoshop 6.0 and then measured using a digital image analysis system (Image‐Pro Plus software, Silver Spring, USA). The bone volume fraction (ratio of bone tissue area to the implant pore area within the implant) was calculated based on Van‐Gieson staining and compared statistically.

##### Immunofluorescent Histochemistry Evaluation of Osteoblasts on the Bone–Implant Interface In Vivo

Four weeks after implantation, rats were sacrificed, and specimens were obtained. After fixation in 10% formalin solution for 4 days, the femurs were first decalcified in 10% ethylenediamine tetraacetic acid (EDTA) (Sigma) solution for 21 days. Using a cutting unit (Leica), 3.5 µm sections were obtained. Sections were processed for standard immunofluorescent histochemical staining and analysis by using primary antibodies, goat anti‐Runx2 (an endothelial cell marker; R&D Systems) and rabbit anti‐OCN (an osteoprogenitor marker; Abcam, Cambridge, England), as well as Alex 594‐conjugated secondary antibodies to corresponding species (Millipore, Billerica, MA). Cell nuclei were stained with 40, 60‐diamidino‐2‐phenylindole (DAPI, Sigma). After being air dried and coverslipped, the sections were observed under a confocal laser scanning microscope (FV‐1000, Olympus, Tokyo, Japan) with the appropriate laser beams and filter settings, and confocal images were captured. For quantitative analysis of the osteoblasts around the implants, a ROI was defined as a ring around the screw extending 200 µm from the lowest point of the thread grooves and barring the region of the screw threads. The area ratio of the total number of Runx2+ and OCN in the ROI was determined with ImageJ software. Four nonadjacent slices of each sample were analysed, with five samples in each group.

##### Immunohistochemical Detection of Proinflammatory and Anti‐Inflammatory Molecules Around Implants

Sections were processed for HE staining, Masson staining, and standard immunohistochemistry analysis with primary antibodies against CD68 (putative macrophages, Abcam), iNOS (a marker of M1 macrophages, Abcam), and CD163 (a marker of M2 macrophages, Abcam). For semiquantification analysis, four slices of each sample were analyzed with three samples in each group. The numbers of cells positive for CD68, iNOS, and CD163 in the ROI around implants were counted.

##### Statistical Analysis

The results are presented as the means ± SD for each group. A one‐way ANOVA followed by Bonferroni's multiple comparison tests was used to perform the statistical analysis. *P*‐values < 0.05 were considered statistically significant.

## Conflict of Interest

The authors declare no conflict of interest.

## Supporting information

Supporting Information
