# Supplementary material for: In Situ Regulation of Macrophage Polarization to Enhance Osseointegration Under Diabetic Conditions Using Injectable Silk/Sitagliptin Gel Scaffolds
Source: Adv Sci (Weinh). 2020 Dec 16;8(3):2002328. doi: 10.1002/advs.202002328 (PMC7856907; doi:10.1002/advs.202002328)
Supplement: Supplementary file 1 — Supporting Information [file ADVS-8-2002328-s001.pdf]

WILEY-VCH

© 2020 Wiley-VCH GmbH

ADVANCED SCIENCE
Open Access

Supporting Information

for *Adv. Sci.*, DOI: 10.1002/advs.202002328

In situ regulation of macrophage polarization to enhance osseointegration under diabetic conditions using injectable silk/sitagliptin gel scaffolds

*Geng Xiang†, Keyin Liu†, Tianji Wang†, Xiaofan Hu, Jing Wang, Zhiheng Gao, Wei Lei *, Yafei Feng*, and Tiger H. Tao**

© 2020 Wiley-VCH GmbH

Supporting Information

Title: In situ regulation of macrophage polarization to enhance osseointegration under diabetic conditions using injectable silk/sitagliptin gel scaffolds

***Authors**: Geng Xiang†, Keyin Liu†, Tianji Wang†, Xiaofan Hu, Jing Wang, Zhiheng Gao, Wei Lei *, Yafei Feng*, and Tiger H. Tao**

G. Xiang, T. Wang, X. Hu, J. Wang, Prof. W. Lei, Y. Feng
Department of Orthopedics, Xijing Hospital, The Fourth Military Medical University, Xi'an, 710032, China
E-mail: Y. Feng: fengyafei2005@163.com; W. Lei: leiwei@fmmu.edu.cn

K. Liu, Z. Gao, Prof. T. H. Tao
State Key Laboratory of Transducer Technology, Shanghai Institute of Microsystem and Information Technology, Chinese Academy of Sciences, Shanghai 200050, China
Email: tiger@mail.sim.ac.cn

Prof. T. H. Tao
Center of Materials Science and Optoelectronics Engineering, University of Chinese Academy of Sciences, Beijing 100049, China

Prof. T. H. Tao
School of Physical Science and Technology, ShanghaiTech University, Shanghai 200031, China

Prof. T. H. Tao
Institute of Brain-Intelligence Technology, Zhangjiang Laboratory, Shanghai 200031, China

Prof. T. H. Tao
Shanghai Research Center for Brain Science and Brain-Inspired Intelligence, Shanghai 200031, China

† These authors contributed equally to this work.

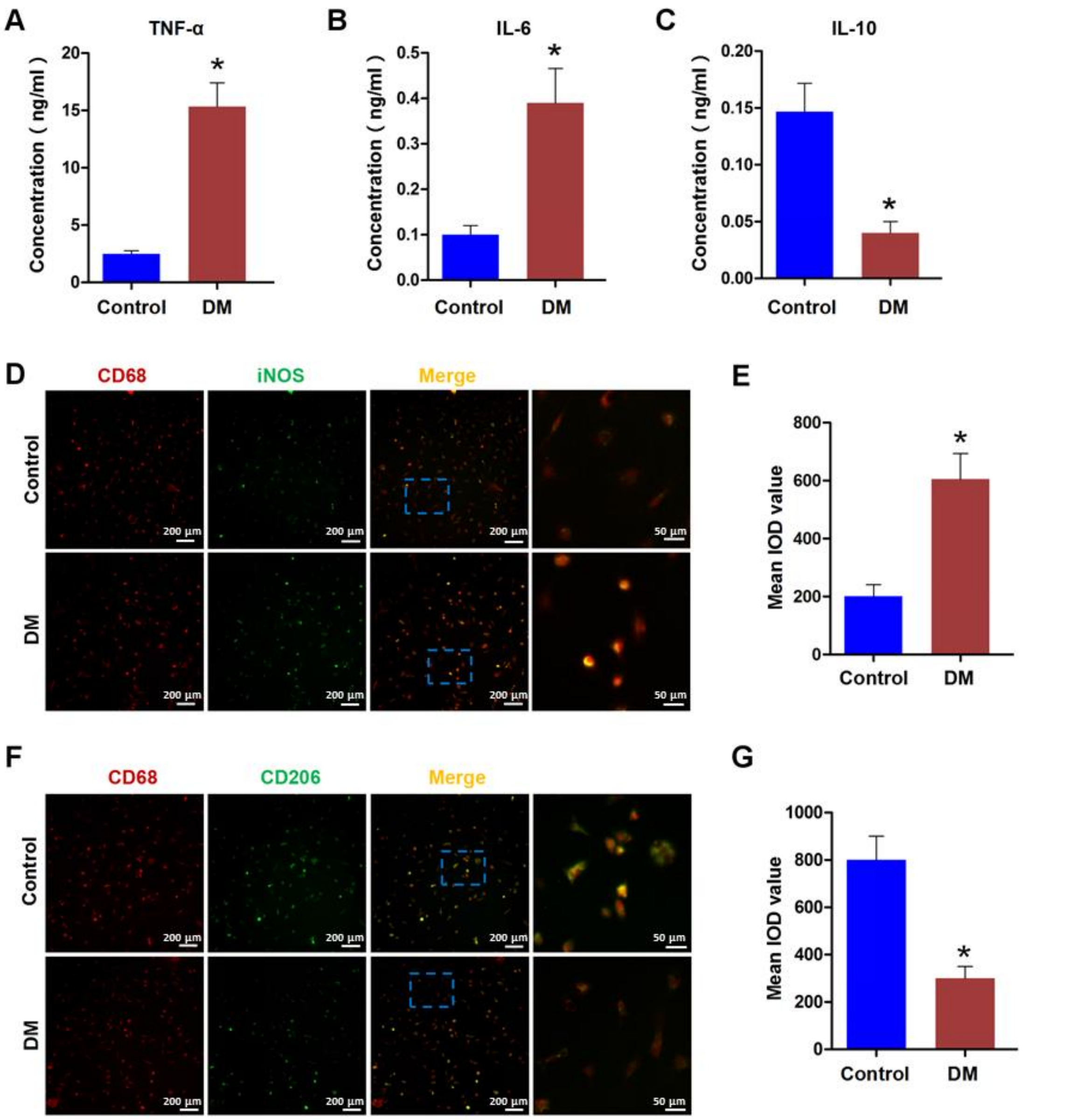

Figure S1. The ELISA analysis of M1/M2 biomarkers and immunofluorescence staining of macrophages in different groups. (A-C) Secretion of the M1 marker TNF-a, IL-6 and M2 marker IL-10 by macrophages. (D, F) Representative images show CD68 (red, marker for macrophages), iNOS (green, marker for M1 macrophages) and CD206 (green, marker for M2 macrophages) expression detected by immunofluorescence staining. (E, G) The semi-quantitative analyses of iNOS and CD206 optical density. IOD, Integrated Optical Density. *$p < 0.05$ vs. Control.

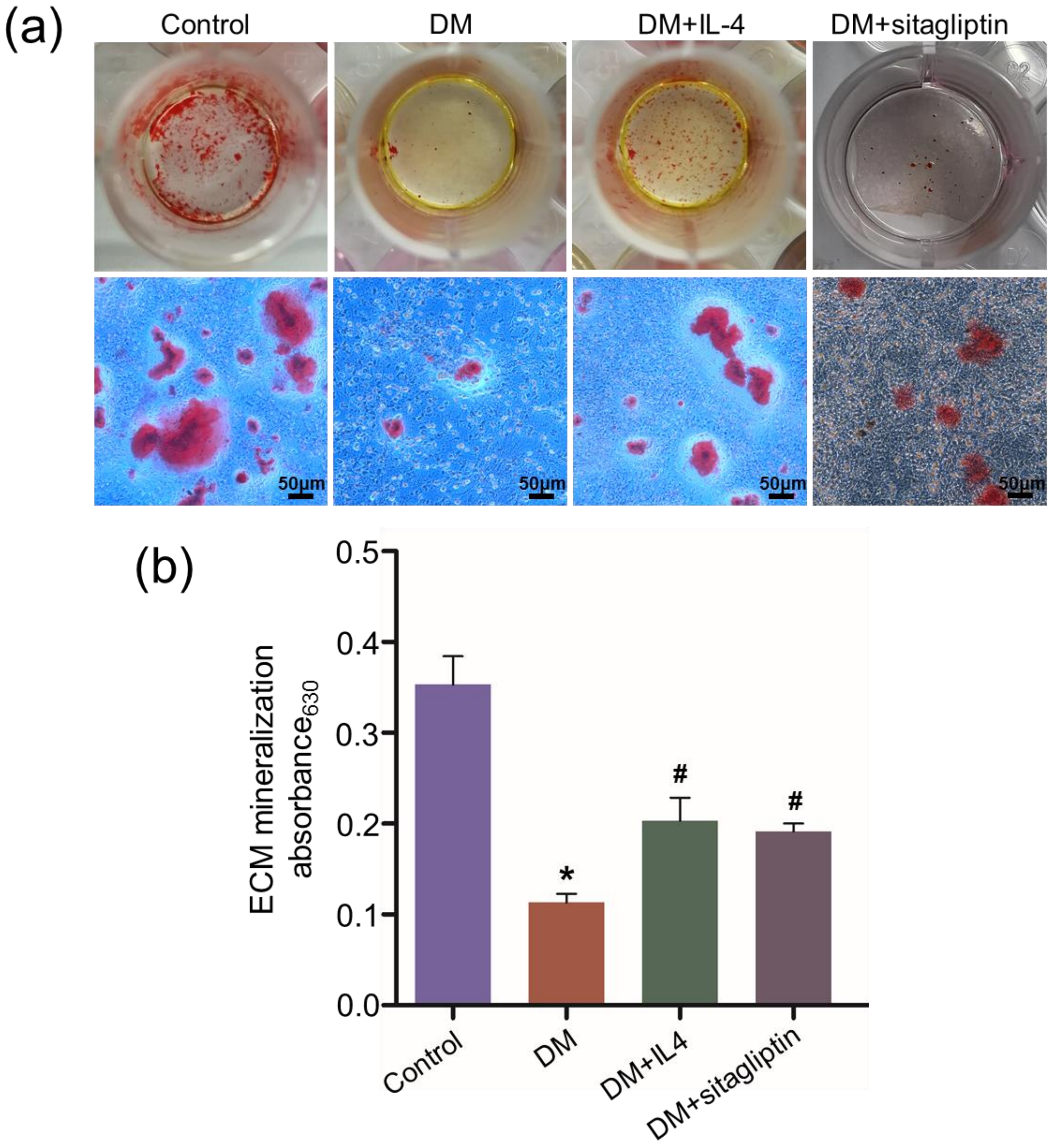

Figure S2. Sitagliptin ameliorated the DM-induced inhibition on the ECM mineralization of osteoblast by repolarizing the macrophage from M1 to M2 phenotypes. (a) ECM mineralization by an alizarin red staining method after 21 days of incubation. (b) Quantitative results of alizarin red staining.*$p < 0.05$ vs. control group; #$p < 0.05$ vs. DM group.

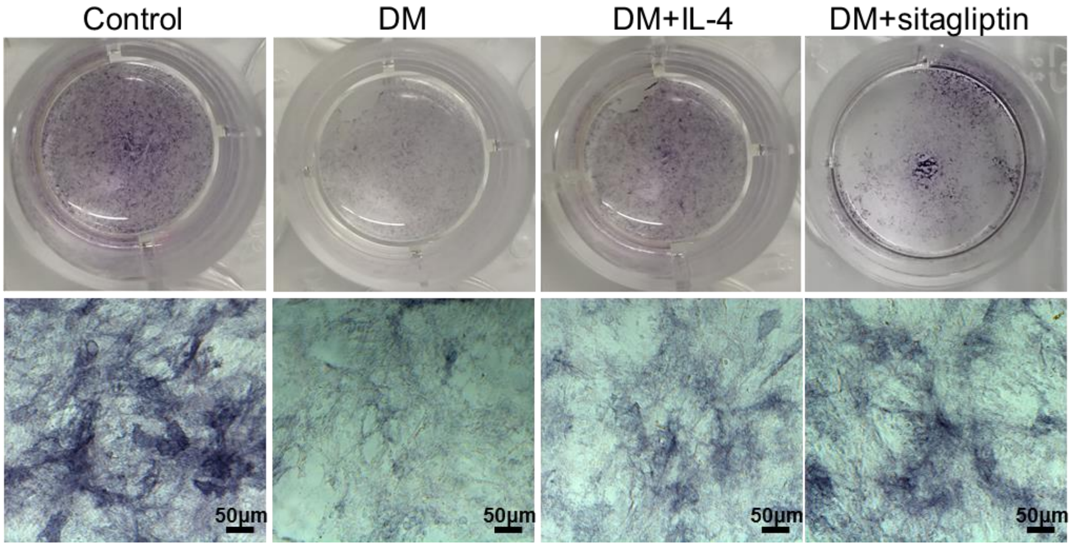

Figure S3. Optical images of ALP-stained osteoblasts after 7 days of incubation.

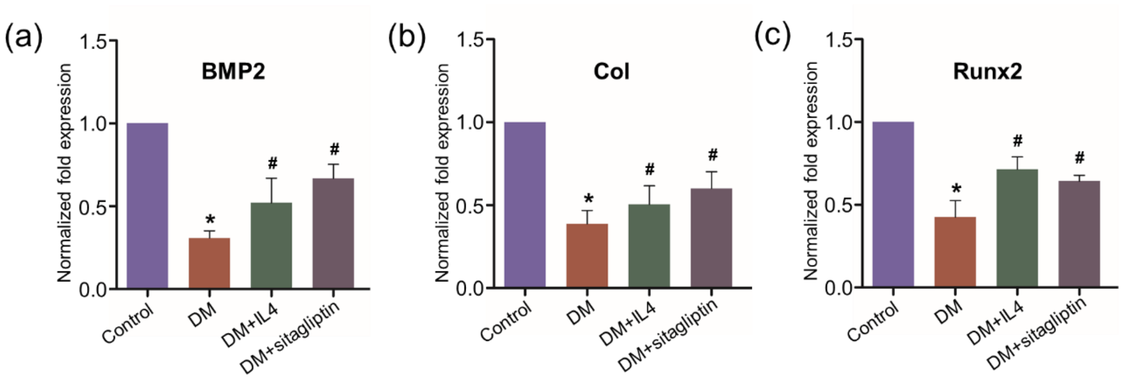

Figure S4. Sitagliptin ameliorated the DM-induced inhibition on the expression levels of the osteogenic genes in transwell co-culture.

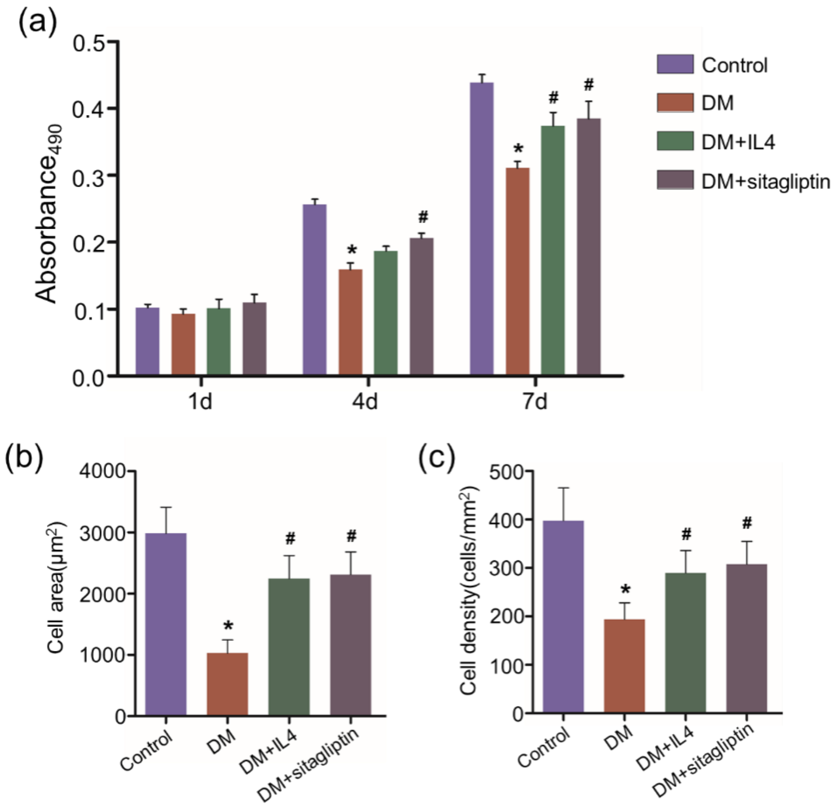

Figure S5. Effects of sitagliptin treatment on the osteoblast proliferation and adhension under diabetic condition. (a) Cell proliferation in different groups after 1, 4 and 7 days of incubation. Histogram of the cell spreading area (b) and cell density (c) in different groups.

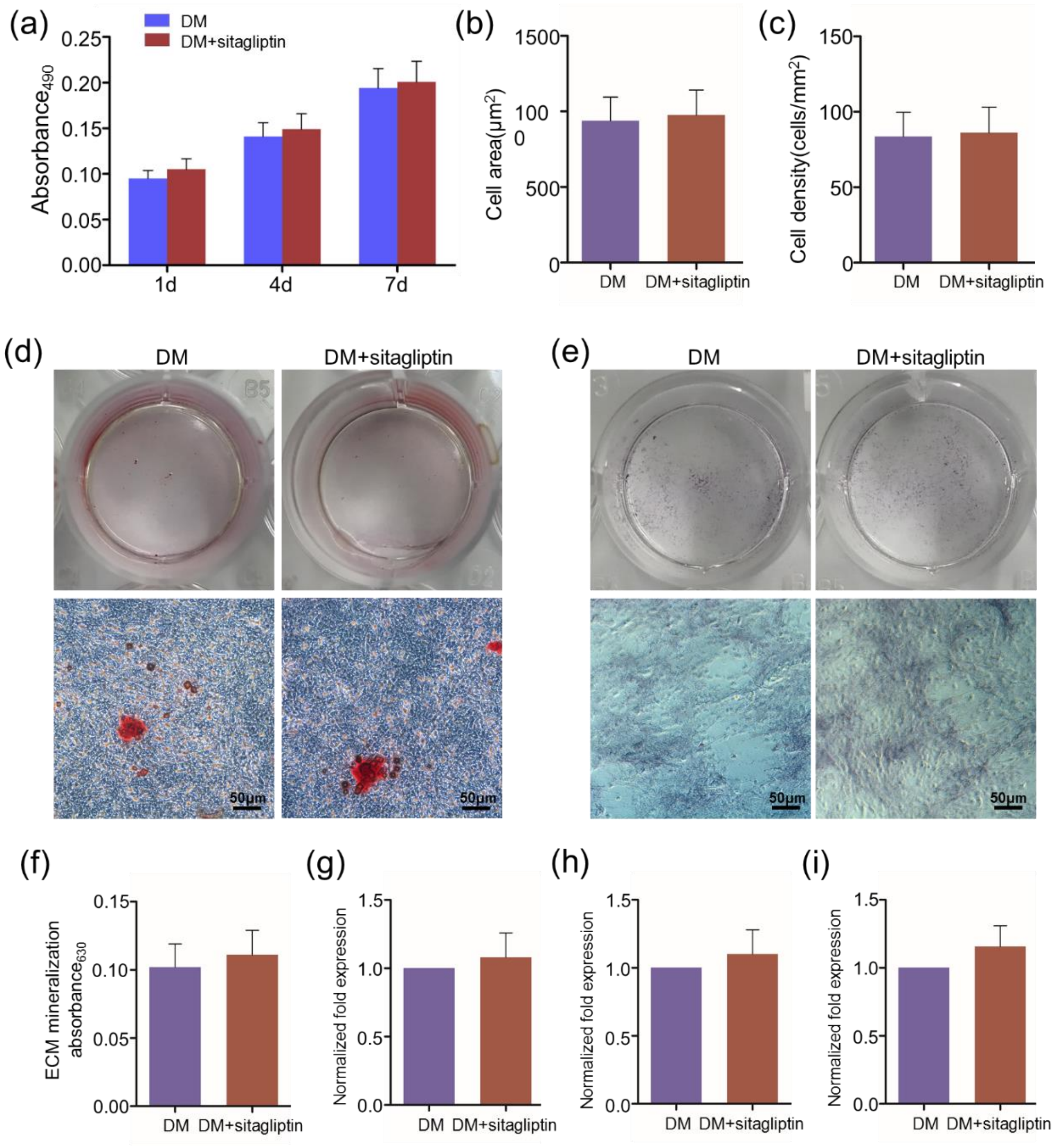

Figure S6. Sitagliptin did not ameliorated the DM-induced inhibition on the biological functions of osteoblast by direct culture. (a) Cell proliferation in different groups after 1, 4 and 7 days of incubation. Histogram of the cell spreading area (b) and cell density (c) in different groups. (d) Typical images of calcium nodes on samples by alizarin red staining after 21 days of incubation. (e) Optical images of ALP-stained osteoblasts after 7 days of incubation. (f) Quantitative analysis of ECM mineralization by the alizarin red staining method. (g-i) Analysis of osteogenic gene expressions with quantitative real-time PCR after 7 days of incubation.

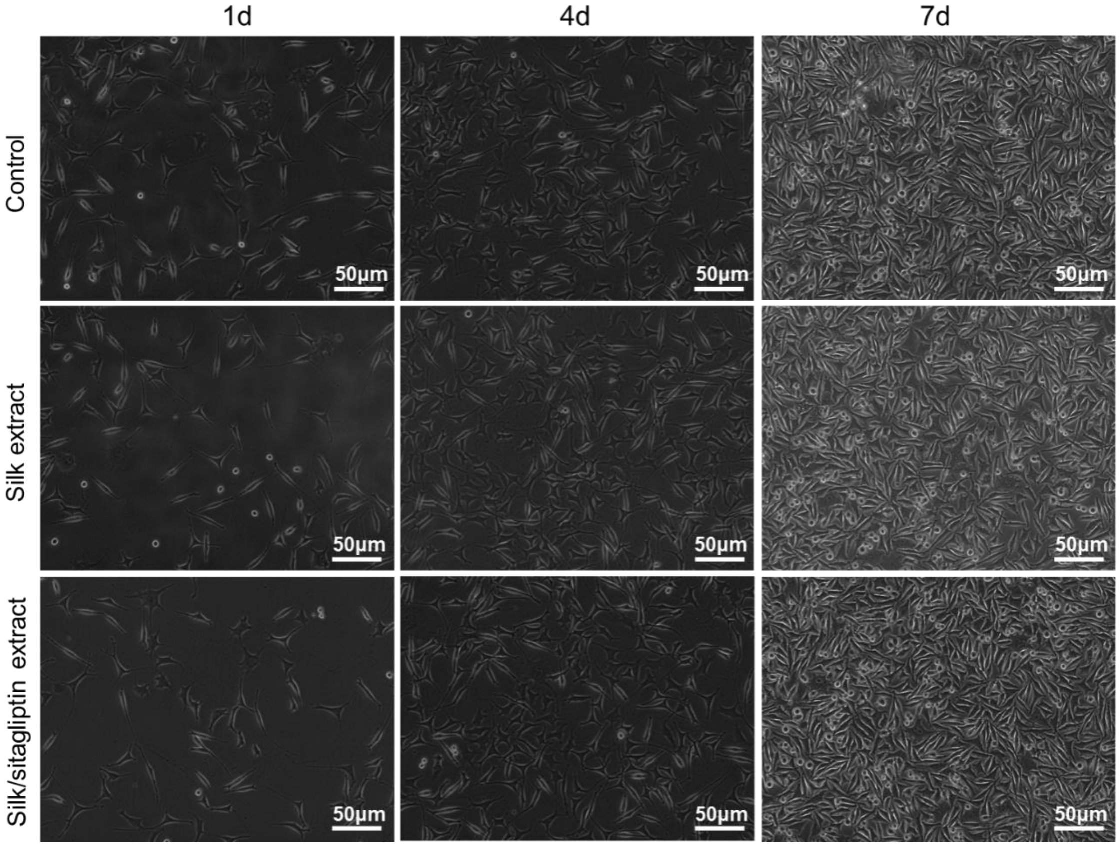

Figure S7. Representative images of L929 cell morphology in different group on 1d, 4d and 7d.

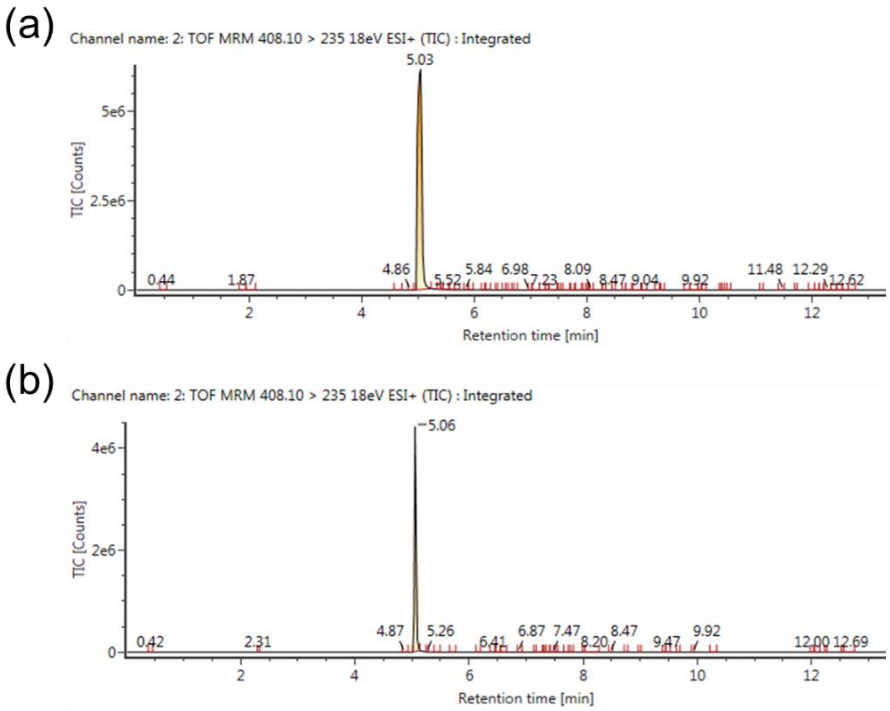

Figure S8. Representative HPLC chromatogram of sitagliptin released from silk/sitagliptin scaffolds after immersing in deionized water: (a) 96h and (b) 1h.

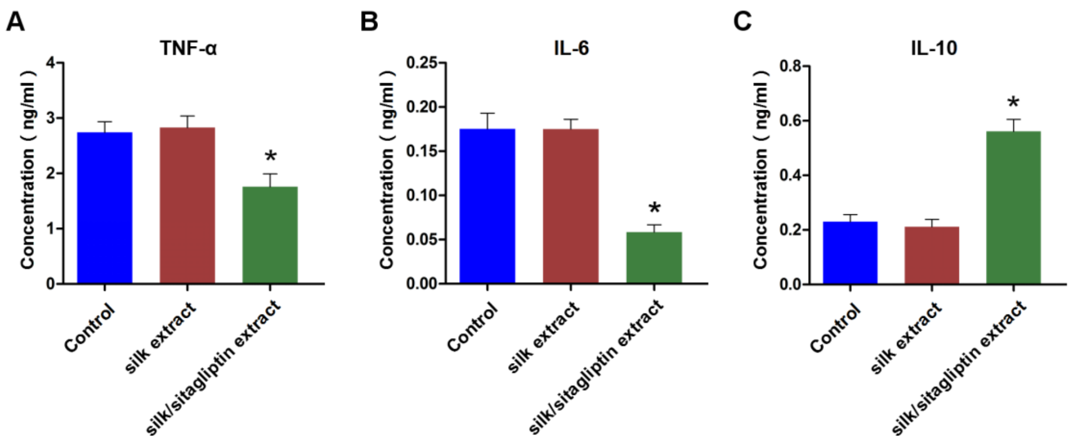

Figure S9. Cytokines secreted by macrophages. Secretion of the M1 marker TNF-a (A), IL-6 (B) and M2 marker IL-10 (C) by macrophages in different groups. **p* < 0.05 vs. Control.

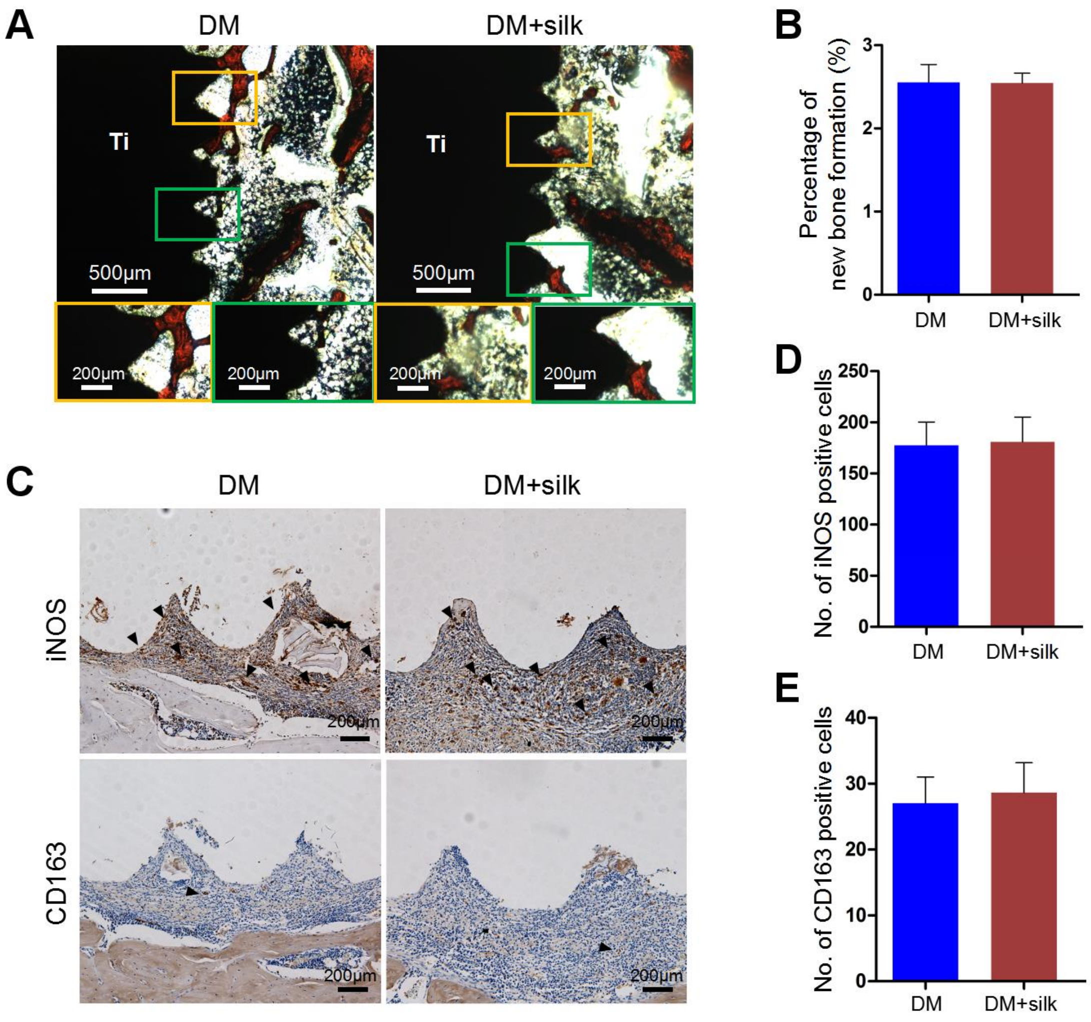

Figure S10. Analysis of new bone formation and macrophage polarization around the implants. (A) Histological images of new bone formation around the implants by Van Gieson staining for newly formed bone (red). (B) Histomorphometric measurement of new bone formation around the implants. (C) Representative immunohistochemical images of iNOS (a marker of phenotype transition caused by M1 polarization) and CD163 (a marker of phenotype transition caused by M2 polarization) at the titanium-bone interface. The numbers of cells positive for iNOS (D) or CD163 (E) in the ROI were counted. *$p < 0.05$ vs. DM group.

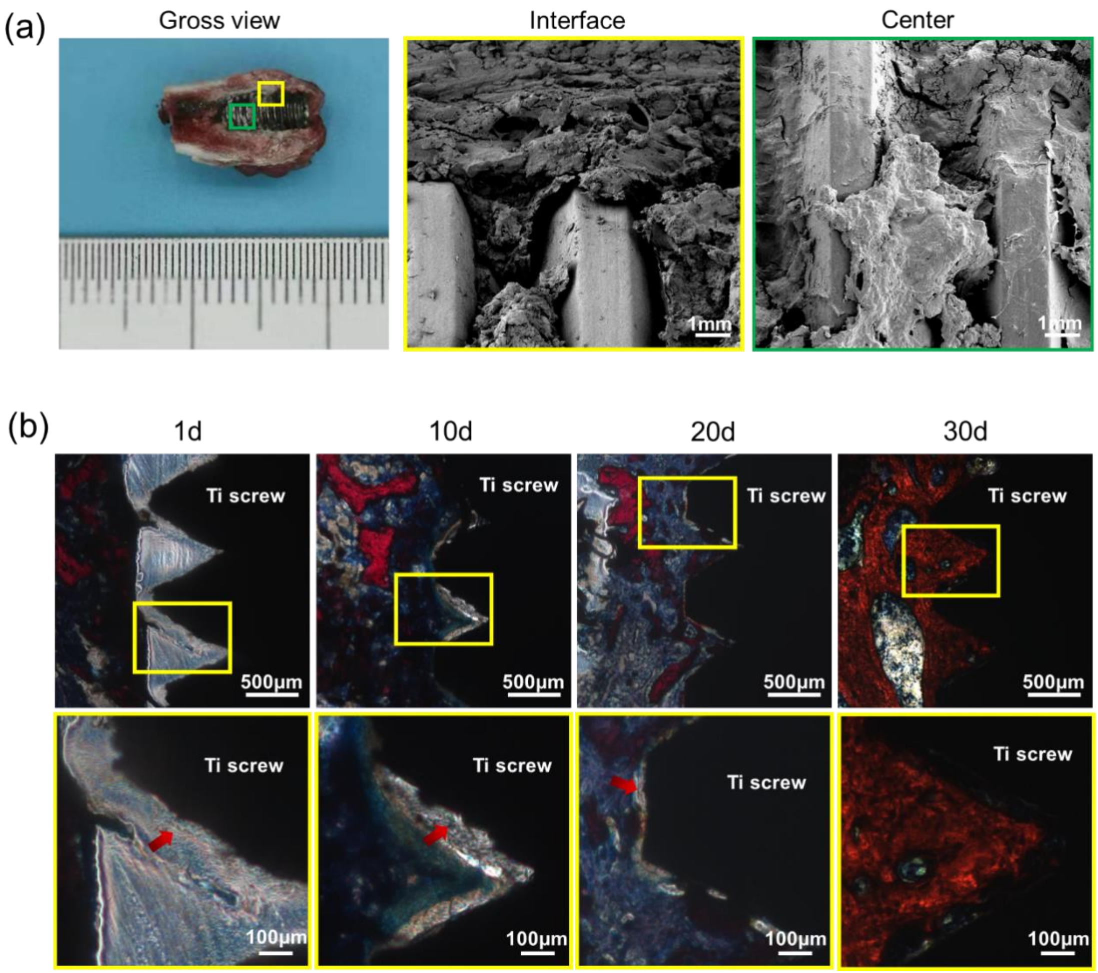

Figure S11. The distribution of silk gel scaffolds around the implant. (a) Cross view and SEM micrographs of silk gel scaffolds around the implant. (b) Histological images of silk gel scaffolds around the implants by Van Gieson staining, the red arrows point to the silk gel scaffolds.

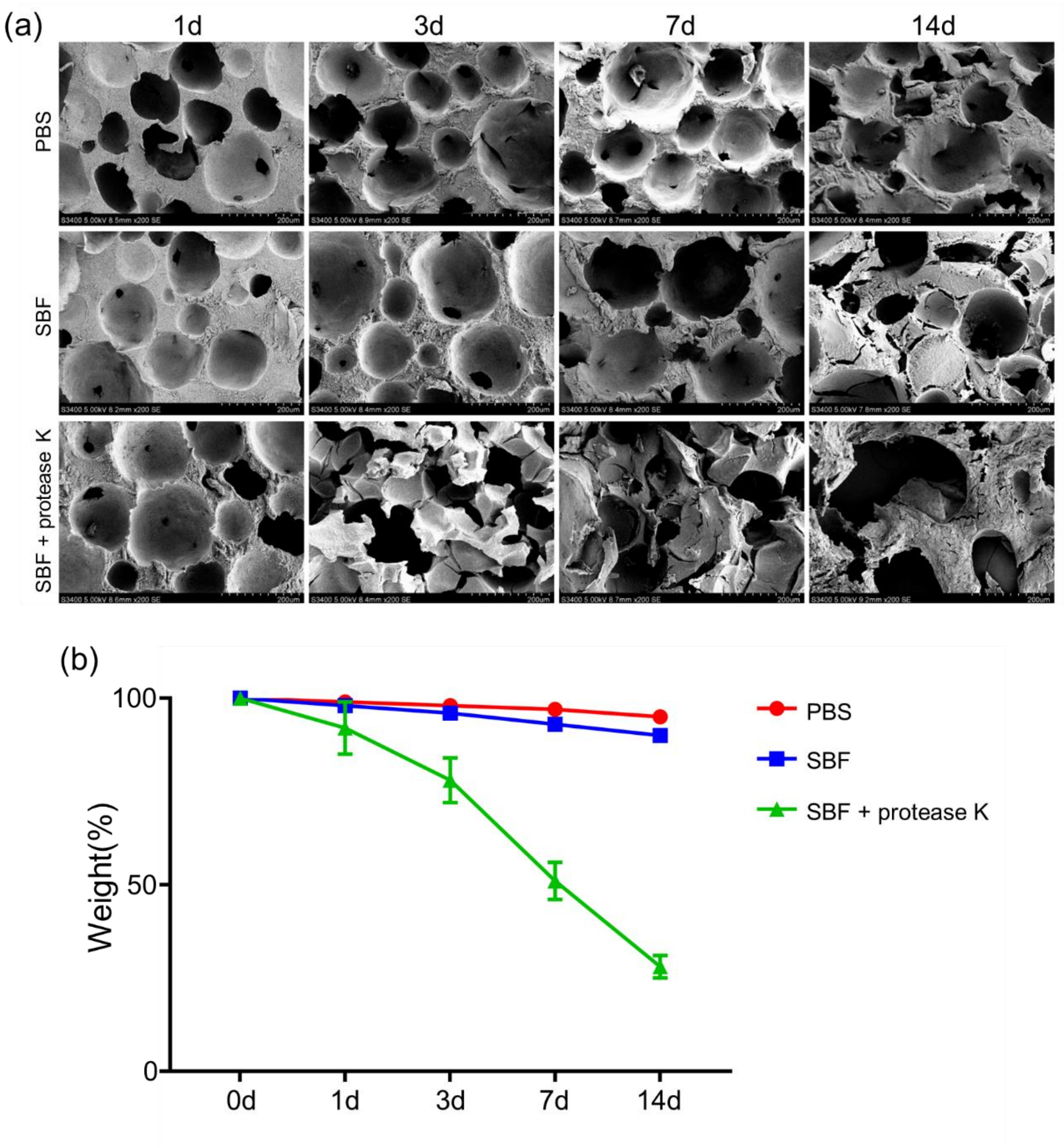

Figure S12. Degradability of silk gel scaffolds in vitro. (a) Representative images of SEM and (b) the percentage of degradation of silk gel scaffolds in different groups on 1d, 3d, 7d and 14d.

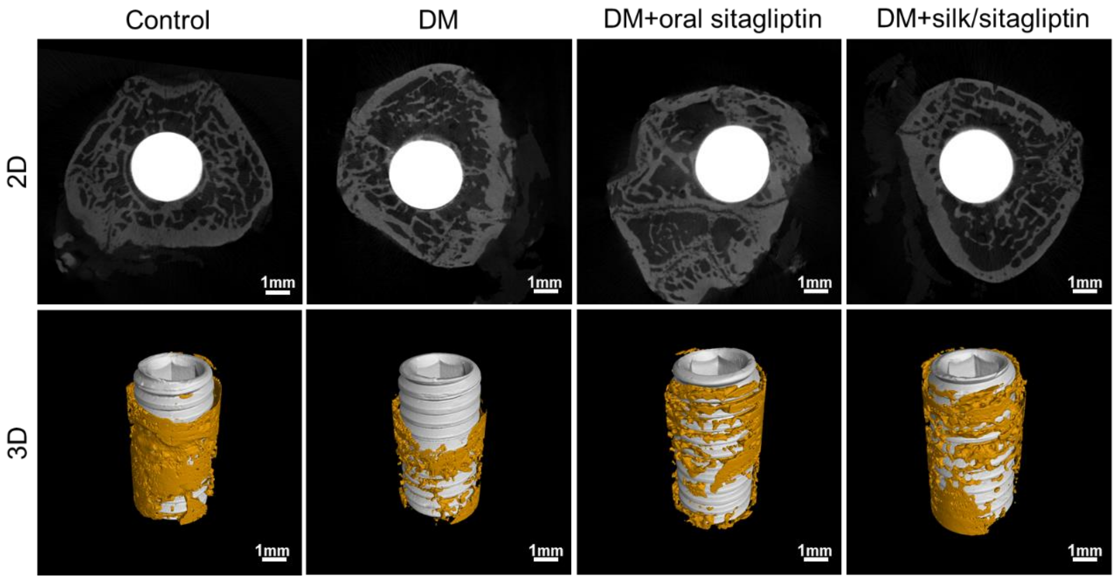

Figure S13. 2D cross-section (upper) and 3D-reconstructed (lower) images in micro-CT analysis showing an in vivo model and the ROI (yellow).

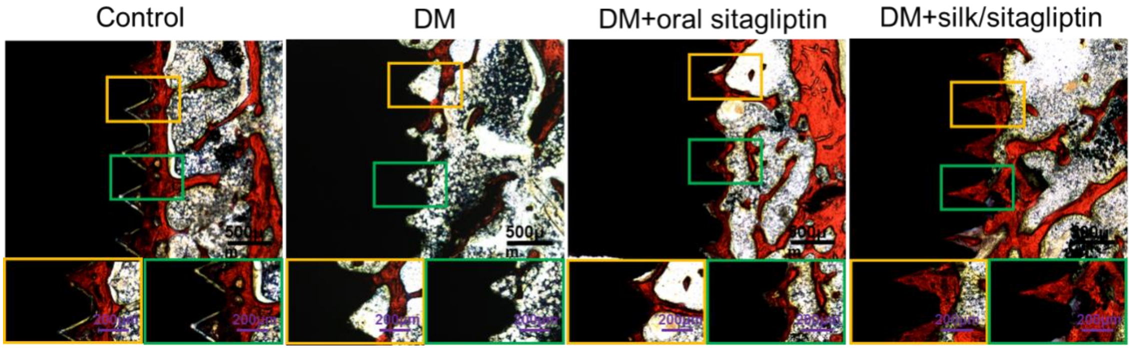

Figure S14. Histological images of new bone formation around the implants by Van Gieson staining. The tissue stained in red color was the newly formed bones.

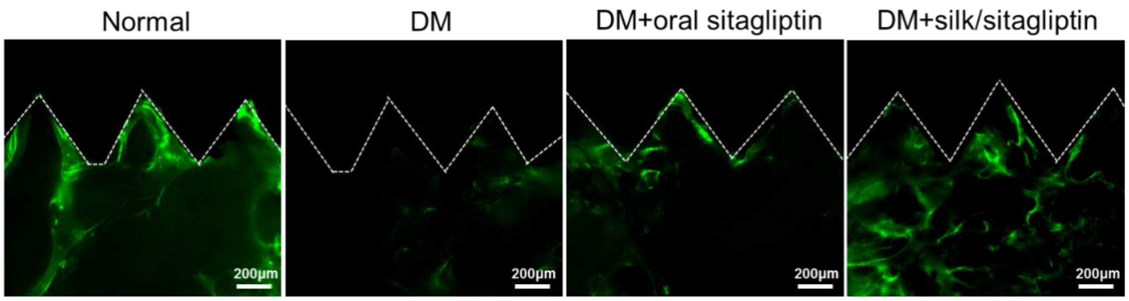

Figure S15. Fluorescent images of histological sections showing new mineral deposition temporally labeled by calcein fluorochrome (green, at 4 weeks).

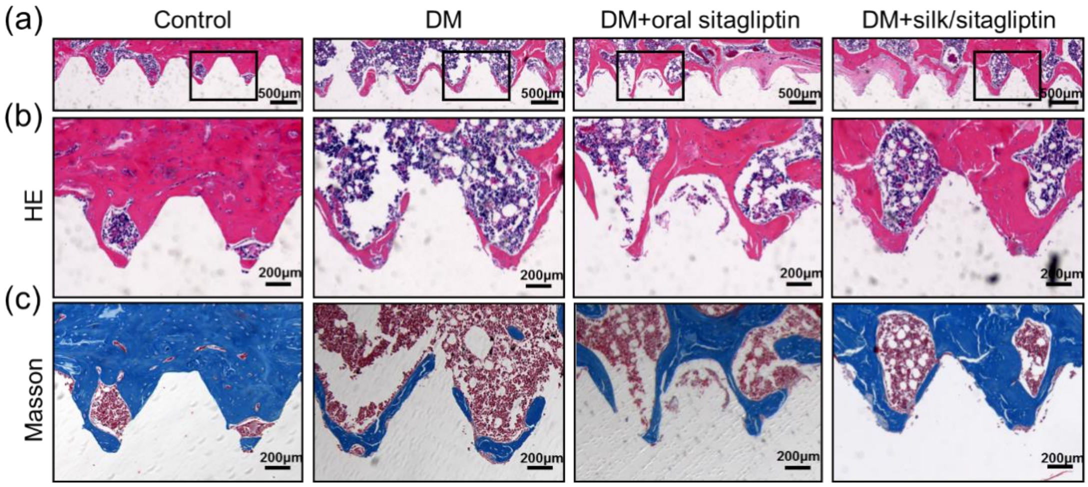

Figure S16. Histological analysis of bone formation around Ti implants in vivo after 4 weeks of implantation. (a-b) Representative HE images of implants from the four groups harvested at week 4. (c) Representative Masson's trichrome staining images showing the expression of collage.

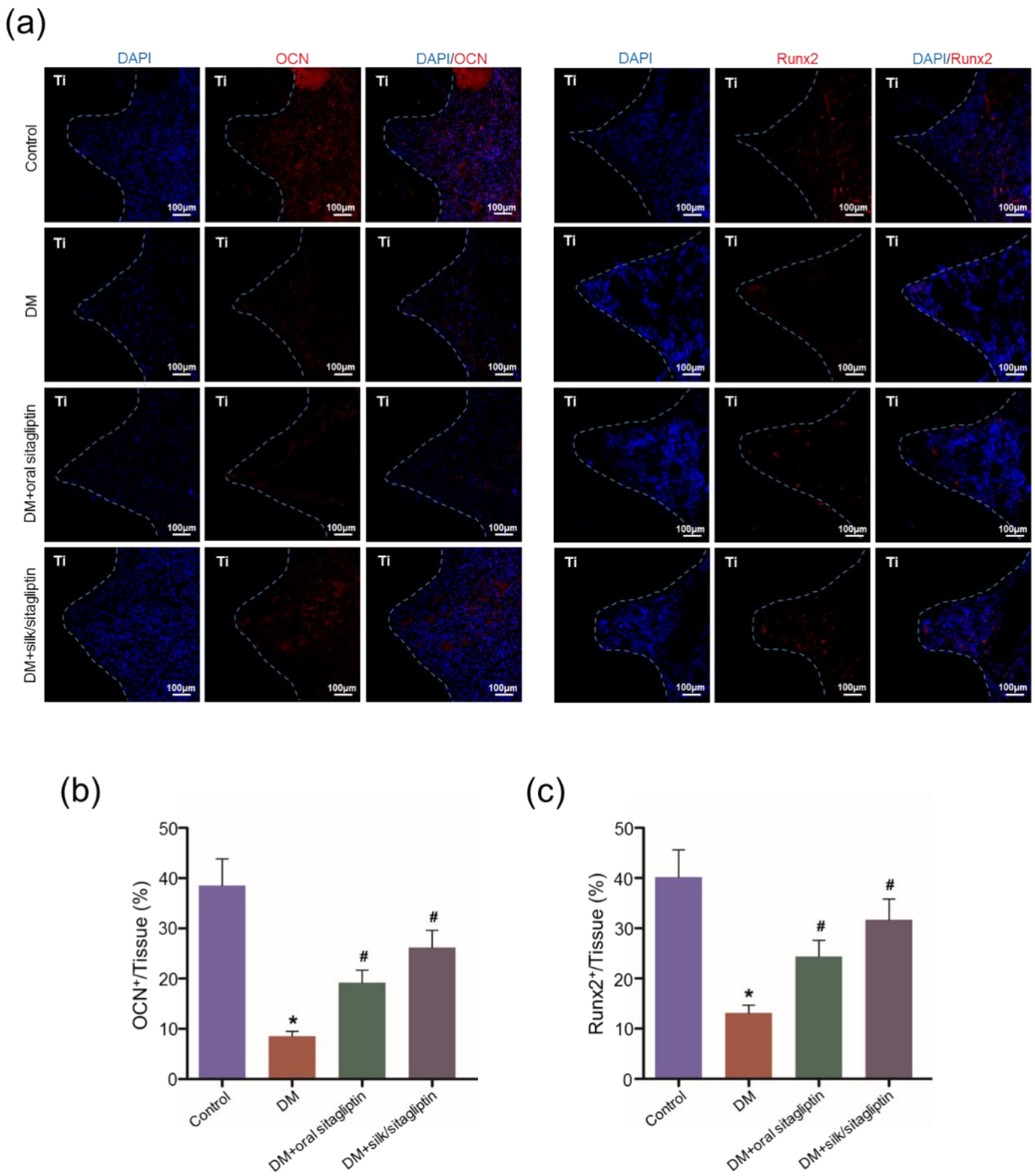

Figure S17. Immunofluorescent staining analysis of bone formation around Ti implants in vivo after 4 weeks. (a) The expression levels of the osteogenic markers Runx2 and OCN around Ti implants after 4 weeks according to immunofluorescent staining. Semi-quantitative results of ratios of (b) OCN or (c) Runx2 positive area to tissue area (Runx2$^+$/Tissue and OCN$^+$/Tissue).

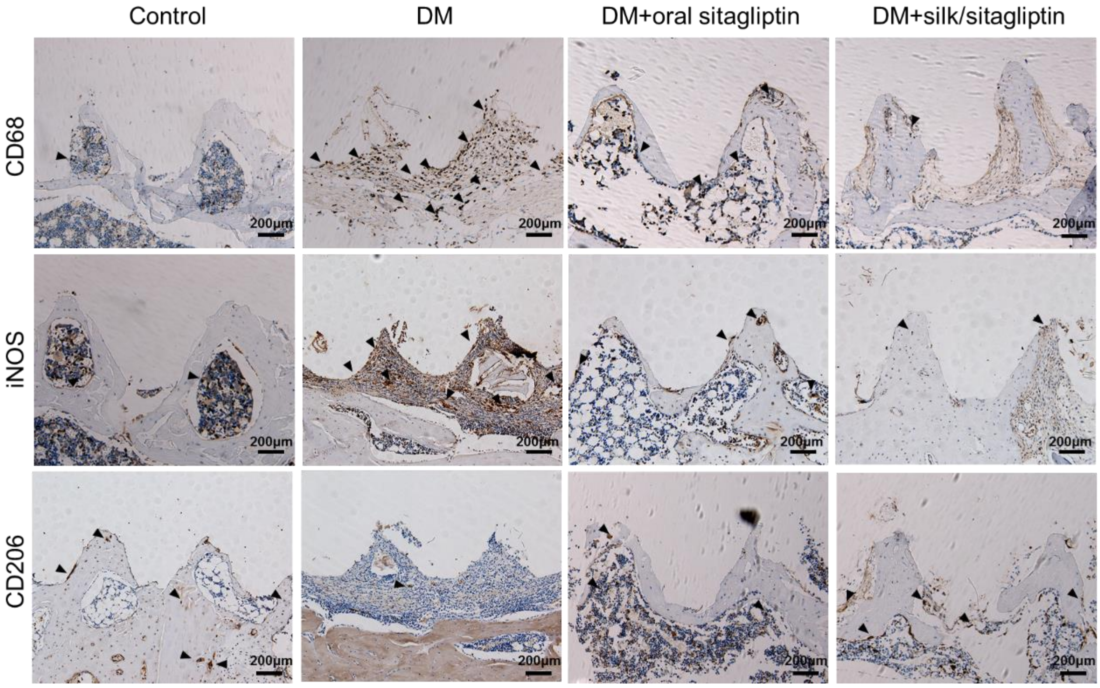

Figure S18. Histological analysis of macrophage polarization around Ti implants in vivo after 4 weeks of implantation.

Table S1. The primers used in the quantitative real-time PCR

Name	Purpose	Sequence
iNOS-F	RT-PCR	5'-GCAGAGATTGGAGGCCTTGTG
iNOS-R	RT-PCR	5'-GGGTTGTTGCTGAACTTCCAGTC
TNF-α-F	RT-PCR	5'-CAGGAGGGAGAACAGAAACTCCA
TNF-α-R	RT-PCR	5'-CCTGGTTGGCTGCTTGCTT
MR-F	RT-PCR	5'-AAACACAGACTGACCCTTCCC
MR-R	RT-PCR	5'-GTTAGTGTACCGCACCCTCC
Fizz1-F	RT-PCR	5'- CGTGGAGAATAAGGTCAAGGAACT
Fizz1-R	RT-PCR	5'- CACTAGTGCAAGAGAGAGTCTTCGTT
Runx2-F	RT-PCR	5'-CACTGGCGCTGCAACAAGA
Runx2-R	RT-PCR	5'-CATTCCGGAGCTCAGCAGAATAA
Col-F	RT-PCR	5'-TCCACATACCTTTATTCCAGGAATC
Col-R	RT-PCR	5'-CCCGGGTTTAGAGACAACTTC
BMP2-F	RT-PCR	5'-CAACACCGTGCTCAGCTTCC
BMP2-R	RT-PCR	5'-TTCCCACTCATTTCTGAAAGTTCC
β-actin-F	RT-PCR	5'-CATCCGTAAAGACCTCTATGCCAAC
β-actin-R	RT-PCR	5'-ATGGAGCCACCGATCCACA
